# Testicular metastasis from urothelial carcinoma: case report and literature review

**DOI:** 10.3389/fonc.2024.1308399

**Published:** 2024-03-14

**Authors:** Changxue Liu, Jing Liu, Qingbo Yang, Leilei Song, Xiaocheng Ma, Yonghua Wang

**Affiliations:** ^1^ Department of Urology, The Affiliated Hospital of Qingdao University, Qingdao, Shandong, China; ^2^ Department of Research Management and International Cooperation, The Affiliated Hospital of Qingdao University, Qingdao, Shandong, China; ^3^ Department of Operating Room, The Affiliated Hospital of Qingdao University, Qingdao, Shandong, China; ^4^ General Office, Urinary Diseases Clinical Medical Research Center of Qingdao, Qingdao, Shandong, China

**Keywords:** urothelial carcinoma, testis, metastasis, chemotherapy, immunotherapy, case report

## Abstract

Urothelial carcinoma (UC) with testicular metastasis is extremely rare, and its modes of metastasis, prognosis, and treatment are unclear. In this report, we present an extraordinarily rare case of testicular metastasis arising from UC 8 years after surgery. The patient underwent left orchiepididymectomy and received immunotherapy postoperatively. After a 6-month follow-up, there were no signs of recurrence. Moreover, the clinical characteristics, metastasis pattern, and treatment plan were also summarized based on 14 earlier reported cases of UC with testicular metastasis.

## Introduction

Urothelial carcinoma (UC) is one of the most common malignant tumors of the urinary system. Bladder tumors, accounting for 90%–95% of UC, are the most frequent urothelial malignancies. Upper urinary tract urothelial carcinoma (UTUC) has a low incidence representing only 5%–10% of all UC cases (1, 2). UC is distinguished by its propensity for multicentric occurrence and recurrence. The most common sites for metastatic spread of UC are the lymph nodes, lungs, liver, bones, and peritoneum (3, 4). Metastatic urothelial carcinoma to the testicle is highly uncommon, and here, we present a case where the tumor originated in the right ureter, spread to the bladder after 3 years, and finally to the left testicle after 5 years. Moreover, we have summarized the clinical characteristics, metastasis pattern, and treatment plan based on the analysis of 14 earlier reported cases of UC with testicular metastasis.

## Case description

**Figure 1:**
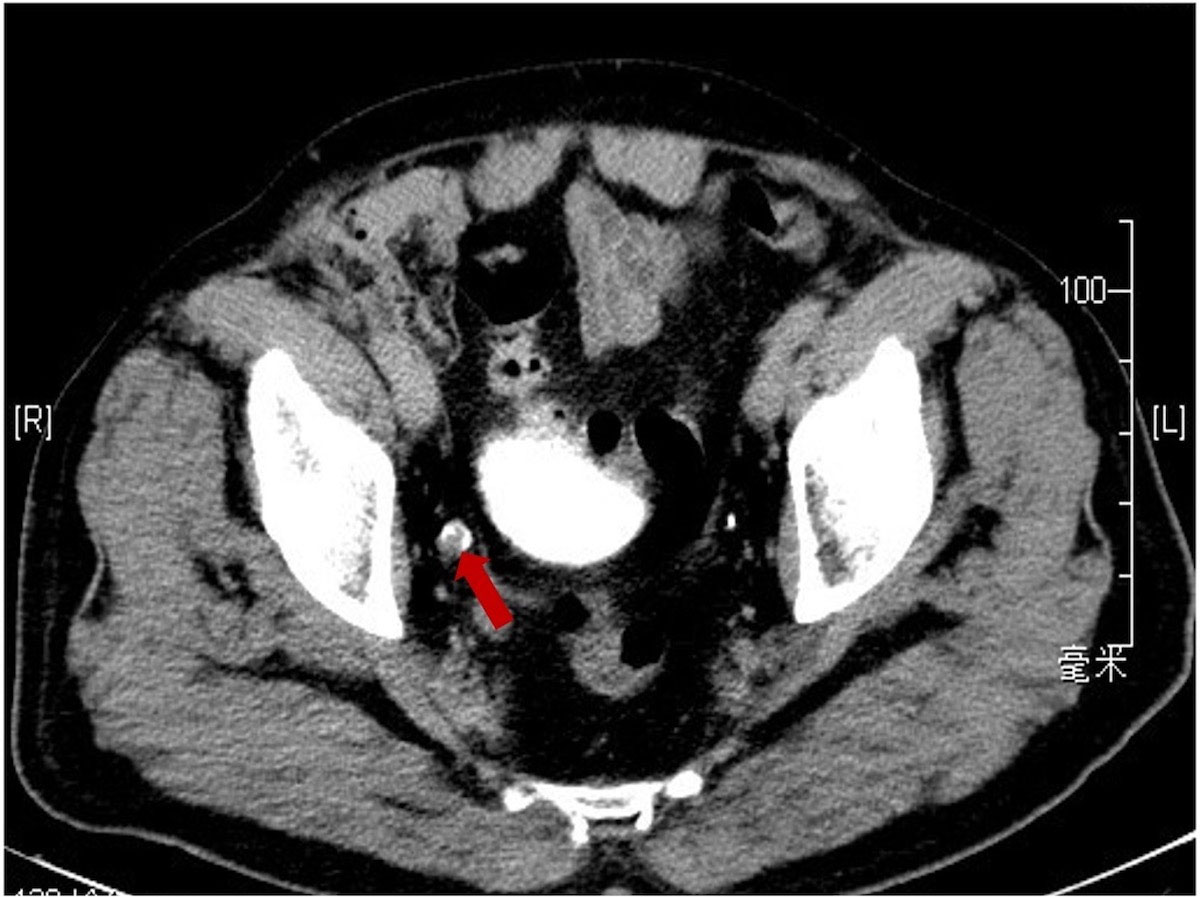
Computerized tomography urography scan showing a soft tissue density shadow (red arrow) in the distal end of the right ureter.

**Figure 2:**
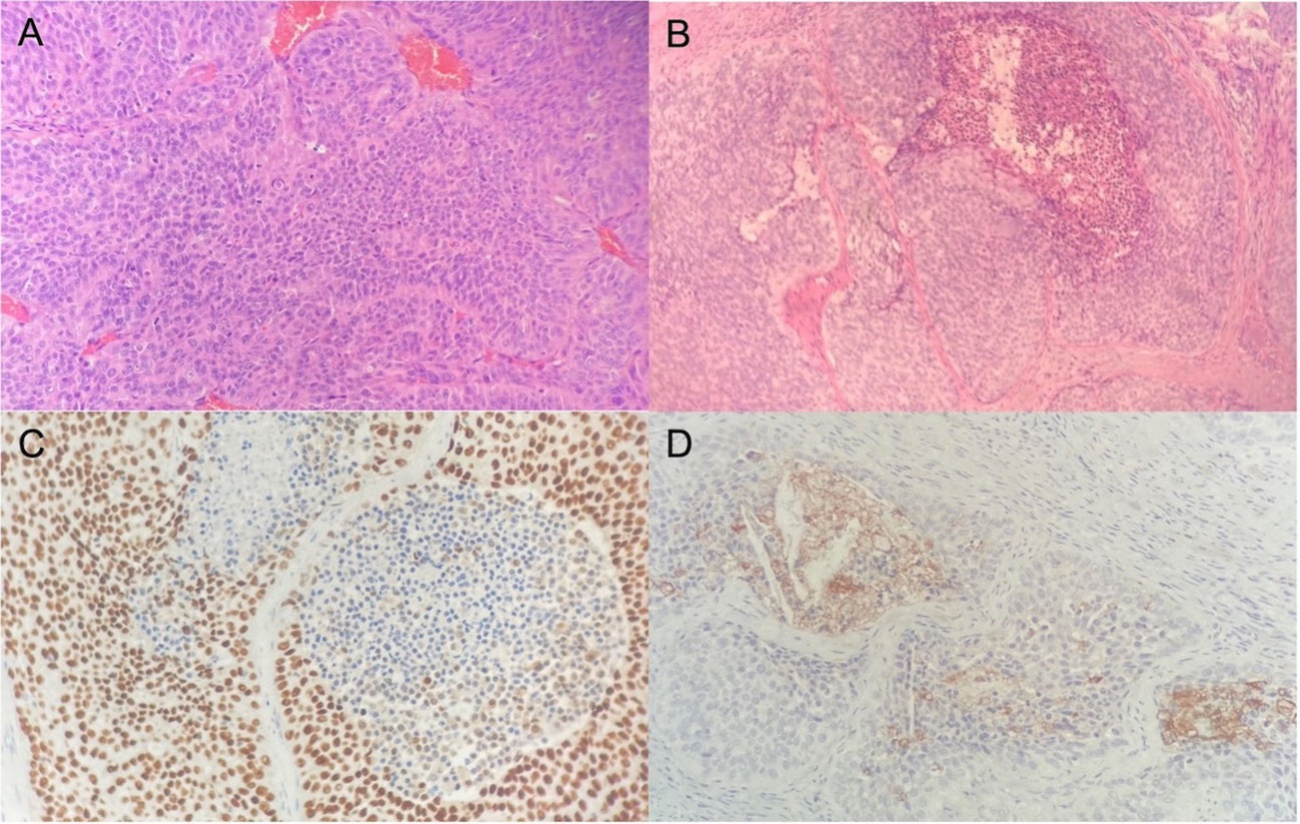
Hematoxylin and eosin stain reveals high-grade urothelial carcinoma in the bladder **(A)** and shows infiltration of urothelial carcinoma in the testis **(B)**. Immunohistochemical illustration of GATA3+ **(C)** and PD-L1+ **(D)** tumor cells.

**Figure 3:**
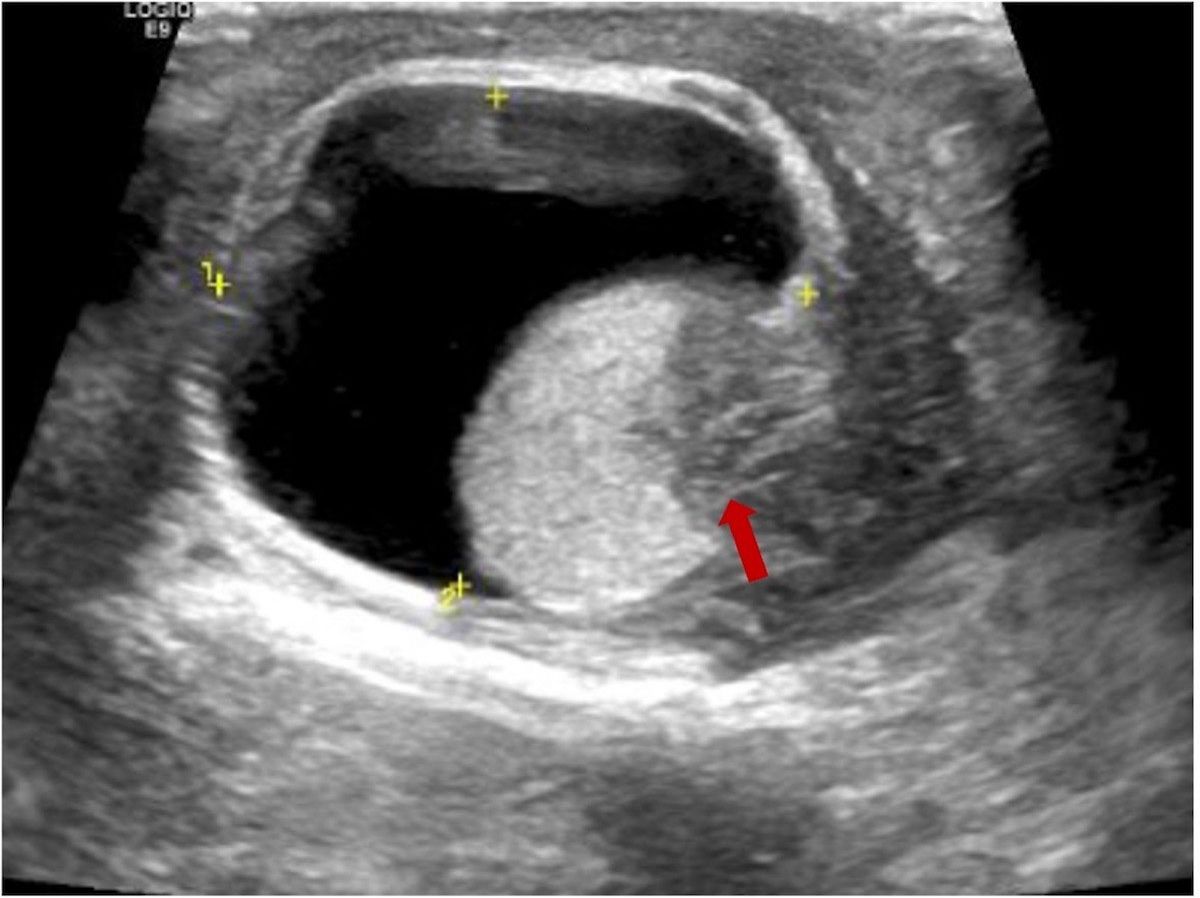
Scrotal ultrasound suggests inflammatory changes in the left epididymis and low echogenicity (red arrow) in the lower part of the left testicle.

The patient was a 75-year-old man with a 40-year history of smoking. In 2014, he came to our hospital for hematuria, which lasted for 10 days. Urinalysis shows positive for occult blood, and computed tomography urography (CTU) scan detected a 1.2-cm-diameter tumor at the end of the right ureter (**Figure 1**). Subsequently, the patient underwent a radical right nephroureterectomy, and postoperative pathology confirmed that the tumor was invasive UC. The neoplasm was staged as G3, pT2N0M0 (WHO 1973) or high grade, pT2N0M0 (WHO 2004). Then, he received an immediate postoperative intravesical instillation of pirarubicin chemotherapy and was followed up regularly for 1 year. In 2017, the patient developed intermittent gross hematuria again. Cystoscopy showed multiple tumors in the bladder, approximately 1.0—1.5 cm in diameter, and histomorphology indicated high-grade UC with carcinoma *in situ* (CIS). The patient subsequently underwent laparoscopic radical cystoprostatectomy, pelvic lymph node dissection, and left ureteral reimplantation surgery. Postoperative pathological examination verified high-grade muscle-invasive UC (**Figure 2A**) with involvement of the prostate and vas deferens. He did not accept additional adjuvant chemotherapy and was followed up regularly for 5 years without tumor recurrence or metastasis. However, until November 2022, the patient had pain and swelling in the left testicle. Scrotal ultrasound suggests a high possibility of epididymitis (**Figure 3**). Anti-inflammatory treatment was given for 1 month without significant improvement, and the left orchiepididymectomy was finally performed. Postoperative pathology suggested high-grade UC, and its histological features were similar to those observed after radical bladder operation (**Figure 2B**). Additionally, immunohistochemical staining showed positive expressions of GATA3 (**Figure 2C**) and PD-L1 (**Figure 2D**) and negative expressions of AFP, PLAP, and Oct-3/4, indicating testicular metastasis from urothelial carcinoma. The patient received tislelizumab immunotherapy every 21 days after surgery and was followed up for 6 months without any signs of tumor recurrence or metastasis.

## Discussion

The typical routes of metastasis in UC include direct invasion, implant spread, lymphatic metastasis, and blood-borne metastasis. Tumors may spread along the urothelium and directly invade the detrusor muscle, ureter, prostate, urethra, uterus, vagina, peribladder fat, intestine, and pelvic lateral wall. Lymphatic metastasis is the route of UC metastasis, whose path varies according to the anatomical location of the tumor. The right UTUC mostly metastasized to the hilum, paracaval, retrocaval, and interaortocaval areas, whereas the left UTUC mostly moved to the hilar and paraaortic regions (5). Besides, the main lymphatic drainage sites of bladder cancer include the internal iliac, external iliac, obturator, and anterior sacral lymph nodes. Advanced urothelial carcinoma may develop hematogenous metastases, which can spread to the liver, lungs, and bones (6). However, UC metastasis to the testicle is extremely uncommon and usually detected at postmortem examination. In one study of cadaveric specimens, the incidence of testicular metastasis was only 0.1% (7), and the primary tumors were prostate, lung, gastrointestinal, melanoma, and renal tumors in descending order of frequency (8).

We retrieved 15 cases reported of UC with testicular metastasis in PubMed from 1984 to the present. As shown in **Table 1**, 10 cases originated from the bladder (6, 8, 10, 12, 13, 15–19), while four cases originated in the upper urinary tract (pelvis, calyceal cavity, and ureter) (7, 11, 14, 20). In the 10 cases that originated from the bladder, seven patients had confirmed invasion of the prostate before the occurrence of testicular metastasis (6, 8, 10, 12, 13, 15, 19). Notably, Dr. Scheldrup proposed that malignant cells from the prostate can extend into the testicular wall along the vas deferens (21) indicating that bladder cancer cells may potentially invade the prostate and then migrate to the testicles along the vas deferens. Furthermore, in the other three cases with no prostate invasion (16–18), transurethral resection of the bladder tumor (TUR-BT) or transurethral resection of the prostate (TURP) were performed two patients, and the risk of tumor cells spreading during surgery cannot be avoided. Thus, tumor metastasis through the vas deferens may be an important mechanism of bladder cancer metastasis to the testis.

**Table 1:** Characteristics of the previously reported cases of urothelial carcinoma with testicular metastasis.

Characteristics of the previously reported cases of urothelial carcinoma with testicular metastasis													
Case	Author	Age	Primary site of urothelial carcinoma	TUR-BT	TUR-P	Radiotherapy	Chemotherapy	Cystectomy	Nephroureterectomy	Tumor stage at diagnosis	Time from initial diagnosis to testicular involvement	Clinical manifestation	outcome
1	Gazouli I et al. (9)	67	Left distal ureter	No	No	Yes	Yes	No	Yes	pT3N2M0	6 months	Left testicular pain	Survival; unknown observation period
2	Fukagawa E et al., (10)	68	Bladder right wall	Yes	Yes	No	No	Yes	No	G3, pT2N0M0	4 years	Left testis painless swelling	Survival; 3 years after orchiectomy
3	Wu X et al. (11)	57	Left Renal pelvis	No	No	No	Yes	No	Yes	pT4NxMx	4 months	Left testicular pain	Died of disease; 1 month after orchiectomy
4	Wu K et al. (12)	67	Bladder neck	No	Yes	Yes	Yes	Yes	No	G3, pT4aN1M0	1 year	Right scrotal pain	Survival; 1 year after orchiectomy
5	Saemundsson Y et al., (13)	37	Bladder	Yes	No	No	Yes	Yes	No	G3, pT4aN1M0	10 years	Left testicular lump	N/R
6	Kubiak M et al. (14)	70	Left renal pelvis	Yes	No	No	No	No	Yes	pT3N0M0	2 years	Left testicular pain	N/R
7	Turo R et al. (15)	61	Bladder floor	Yes	No	No	No	Yes	No	G3, pT4N0M0	2 years	Right painless testicular swelling	N/R
8	Manav A et al. (7)	56	Left proximal ureter	No	No	No	No	No	No	N/A	0	Left testicular swelling	N/R
9	Kiely G et al. (16)	71	Bladder	Yes	No	Yes	Yes	No	No	G3, pT2N0M1	1 year	Painless swelling of both testicles (left first, then right)	Died of disease; 2 years after initial presentation
10	Kozak G et al. (17)	84	Bladder anterior wall	Yes	Yes	No	No	No	No	pT1NxMx	1 year	Right testicular hard	N/R
11	Mahmalji W et al. (6)	72	Bladder left wall	Yes	Yes	No	No	Yes	No	G2, pT4aN0M0	10 years	Left testicular pain	Survival; unknown observation period
12	Thwaini A et al. (18)	74	Bladder	Yes	No	No	No	Yes	No	G2, pT3N0M0	3 years	Left testicular painless swelling	N/R
13	Morgan K, et al. (8)	74	Bladder posterior wall	Yes	Yes	Yes	Yes	Yes	No	N/A	Synchronous	Right testis hard and painful	Survival; 1.5 years after adjuvant chemotherapy
14	Binkley,W et al. (19)	69	Bladder triangle	Yes	Yes	Yes	No	No	No	pTxN1M0	8 months	Left testis tender and slightly enlarged	Died of disease; 8 months after orchiectomy
15	Our case	75	Right distal ureter	No	No	No	No	Yes	Yes	G3, pT2N0M0	8 years	Left scrotum swollen and hard	Survival; unknown observation period

G, grade; N/A, not available; N/R, not reported.

Interestingly, in four cases originating from the upper urinary tract, the patients had primary left UTUC and eventually spread to the left testicle (7, 11, 14, 20). Anatomically, the right testicular vein drains into the inferior vena cava, while the left testicular vein drains into the left renal vein (9). Due to the reverse reflux transfer mechanism, tumor cells can travel through blood vessels and lymphatics. Thus, tumor cells from the left upper urinary tract could metastasize to the left testicle through the reverse flow from the left renal vein to the left testicular vein, which may be an important mechanism of UTUC metastasis to the testis. In our case, although the primary tumor was located at the distal end of the right ureter, metastasis ultimately occurred in the contralateral testis. We suspect that this may have been due to implantation metastasis of the ureteral tumor leading to bladder cancer, followed by invasion of the prostate and vas deferens. Therefore, we suspect that this patient experienced testicular metastasis through the vas deferens pathway.

Another note is that testicular metastasis in UC may present as testicular enlargement with accompanying symptoms of epididymitis or asymptomatic testicular enlargement, which may increase the risk of misdiagnosis. Testicular magnetic resonance imaging can aid in differential diagnosis, and if necessary, biopsy or excision may be performed.

Since the late 1980s, combination chemotherapy containing cisplatin has been the first-line standard treatment for metastatic urothelial carcinoma (UC), with common regimens including MVAC and GC (22). In our case, post-orchiectomy histopathology confirmed vascular invasion. Consequently, we planned to initiate chemotherapy using the GC regimen. However, the patient, who was concerned of the significant side effects of chemotherapy, declined the proposed treatment. In recent years, an increasing number of immune checkpoint inhibitors have been approved for clinical use showing promising therapeutic effects in solid tumors, including UC. Based on the results of two single-arm phase II trials, the US Food and Drug Administration (FDA) and the European Medicines Agency (EMA) have sanctioned the use of immune checkpoint inhibitors as a first-line treatment for cisplatin-ineligible patients with PD-L1-positive tumors (22). In our case, despite the patient’s refusal of chemotherapy, immunohistochemistry results from the testicular metastatic lesion revealed positive PD-L1 expression. Consequently, we opted for tislelizumab immunotherapy, and the patient remained free of tumor recurrence or signs of metastasis during the 6-month follow-up. However, to date, the efficacy of immune checkpoint inhibitors in patients with testicular metastasis from UC has not been confirmed in prospective trials with evidence limited to individual case reports. Gazouli et al. reported a case of UTUC with testicular metastasis treated with pembrolizumab postoperatively, but disease progression was observed despite immune checkpoint inhibitor administration (20). Therefore, identifying suitable biomarkers to predict the efficacy of immune checkpoint inhibitors is crucial. Relying solely on PD-L1 positivity may be insufficient due to variations in antibodies and scoring systems. Research suggests that high tumor mutation burden (TMB) is another biomarker for predicting immune therapy response (23), but further validation in randomized clinical trials is necessary. In conclusion, the effectiveness of postoperative immune-assisted therapy and reliable predictive biomarkers in patients with UC and testicular metastasis warrant further investigation.

## Conclusions

We report a rare case of metastatic urothelial carcinoma of the testicle and discuss the clinical features, metastasis mechanism, and treatment of metastatic urothelial carcinoma of the testicle based on a literature review. Although testicular metastasis of UC is rare, clinicians should be aware of the possibility of metastasis to the testicle in patients with a history of UC with testicular symptoms.

## Data availability statement

The original contributions presented in the study are included in the article/supplementary materials, further inquiries can be directed to the corresponding author/s.

## Ethics statement

The studies involving humans were approved by The Medical Ethics Committee of the Affiliated Hospital of Qingdao University. The studies were conducted in accordance with the local legislation and institutional requirements. The human samples used in this study were acquired from a by-product of routine care or industry. Written informed consent for participation was not required from the participants or the participants’ legal guardians/next of kin in accordance with the national legislation and institutional requirements. Written informed consent was obtained from the individual(s) for the publication of any potentially identifiable images or data included in this article.

## Author contributions

CL: Writing – original draft, Project administration, Investigation, Conceptualization. JL: Writing – review & editing, Conceptualization. QY: Writing – review & editing. LS: Writing – review & editing. XM: Writing – review & editing, Supervision, Conceptualization. YW: Writing – review & editing, Supervision, Conceptualization.
